# Large spontaneous lung hernia—a case report

**DOI:** 10.1093/jscr/rjad534

**Published:** 2023-12-07

**Authors:** Hareshdeva Devan Nair, Bibhusal Thapa, Krishna Bhagwat

**Affiliations:** Department of Thoracic Surgery, Northern Hospital, 185 Cooper St, Epping, Victoria, VIC 3076, Australia; Department of Thoracic Surgery, Northern Hospital, 185 Cooper St, Epping, Victoria, VIC 3076, Australia; Department of Thoracic Surgery, Northern Hospital, 185 Cooper St, Epping, Victoria, VIC 3076, Australia

**Keywords:** chronic obstructive pulmonary disease, cough, smoking, spontaneous lung herniation

## Abstract

Intercostal lung hernias can be results of trauma or surgery. True spontaneous lung hernias thought to be a result of sudden acute increase in intrathoracic pressures are extremely rare. Only 51 cases have been reported in literature over the past four-and-half decades. We report a case of spontaneous chest wall posterior-lateral lung herniation that developed in a 73-year-old following severe coughing and straining during an episode of lower respiratory tract infection. Herein we present the unusual findings in the case and outline the pathophysiology of the problem and management options available.

## Introduction

Lung herniation is defined as a protrusion of lung parenchyma outside of the thoracic cavity through a defect in the chest wall or diaphragm [1]. The majority of lung hernias are acquired, whereas only one in every five occurrences is the result of congenital anomalies [2]. Trauma and preceding operative procedures are the most common causes for lung herniation. However, a small fraction of acquired lung hernias in whom there is no history of surgery or thoracic trauma are referred to as ‘spontaneous’ lung hernias. These lung hernias generally result from events leading to a sudden increase in intrathoracic pressure such as coughing, sneezing, or heavy lifting with subsequent rupture of the intercostal muscles or even rib fracture [2]. On account of the small number of cases that have been reported there have only been a few speculations about the potential risk factors involved in the causation. Chronic Obstructive Pulmonary Disease (COPD) is a frequent comorbidity in patients with a spontaneous lung herniation. Presumably, chronic coughing and hyperinflation of the lung, perhaps combined with long-term steroid administration may be attributed to the development of the hernia [3, 4]. Male gender, smoking history, and obesity might be other risk factors [2].

The usual symptomatology consists of pain which increases with coughing or straining. Chest wall ecchymosis and a soft, bulging, subcutaneous mass that protrudes from the chest wall and enlarges with the Valsalva maneuvre, coughing, or physical strain is evident upon examination. A computer tomography (CT) scan of the chest usually confirms the diagnosis [5].

Although conservative observational management [6] and non-operative treatment by thoracic strapping have been described and practised in the past [7], development of respiratory distress, an increase in pain or size of the herniation, impending incarceration, or difficulty to reduce the hernia necessitate operative repair which can be achieved with excellent results and low morbidity [2, 3].

## Case report

A 73-year-old male presented with a history of sudden right upper quadrant abdominal pain, swelling, and bruising in the right lateral chest wall secondary to recurrent episodes of coughing from a lower respiratory tract infection. He was a retired bricklayer with a past medical history of hypertension, atrial fibrillation on anticoagulation, dyslipidemia, obesity, obstructive sleep apnea, and arthritis. He had no surgical history and was a non-smoker.

**Figure 1:**
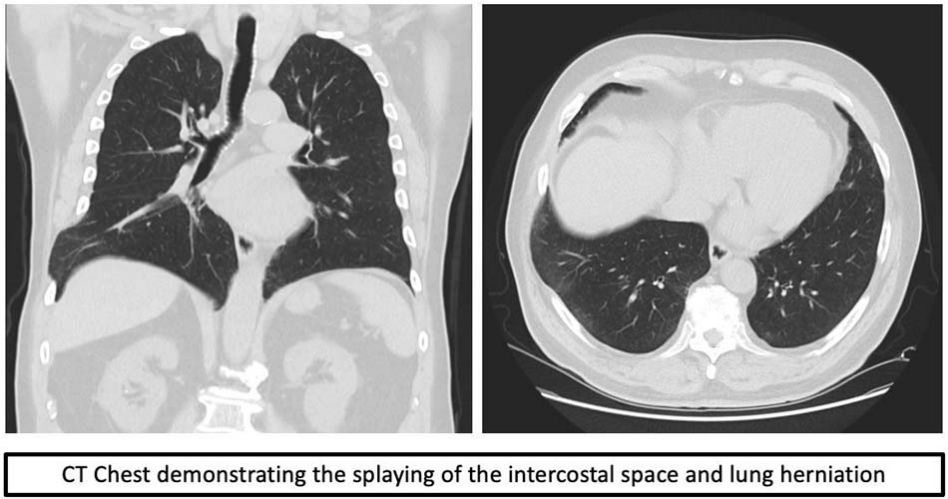
CT scan.

Over time, the bruising subsided, however he continued to have a bulge and localized pain. A CT scan of the chest revealed splaying of the eighth and ninth ribs with bulging of lung and perihepatic fat consistent with herniation (Fig. 1). He was referred to Thoracic surgery outpatient clinic for consideration of surgical management due to ongoing symptoms.

After initial evaluation, the patient proceeded to operative repair. The operation was performed under general anesthetic with double lumen endotracheal tube and right-sided lung isolation.

**Figure 2:**
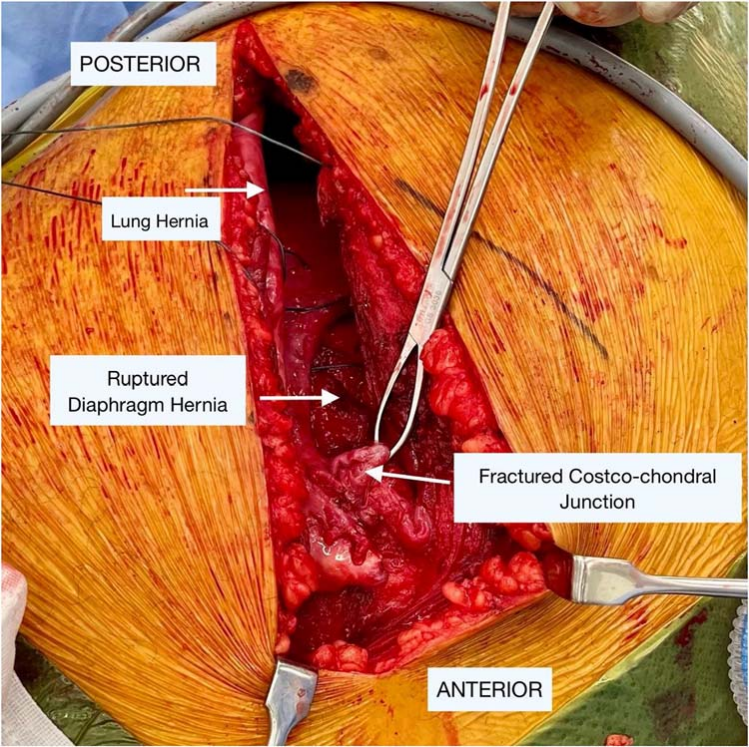
Intraoperative findings of the lung and diaphragmatic herniae as well as the fractured costochondral junction as labeled in image.

An incision was made directly over the palpable defect in the lower anterolateral chest. The intercostal muscles of the eighth space were found to have been ruptured and splayed throughout the entire length of the space with a clearly identifiable fracture dislocation of the costochondral junction of the eighth rib. Interestingly, there was also a rupture of the anterolateral aspect of the diaphragm with herniation of part of liver into the chest. The parietal pleura was ruptured and it formed part of the hernia sac. There were adhesions of the liver to the lung parenchyma (Fig. 2).

After adhesiolysis, the diaphragmatic defect was repaired using double layer continuous and interrupted 2.0 prolene sutures and was also reinforced with a prolene mesh (ETHICON, Johnson & Johnson, New Brunswick, NJ, USA). The eighth intercostal space was closed with multiple figures of eight sutures using five Ethibond suture (ETHICON, Johnson & Johnson, New Brunswick, NJ, USA). The muscles, subcutaneous layers, and skin were closed in layers in routine fashion.

**Figure 3:**
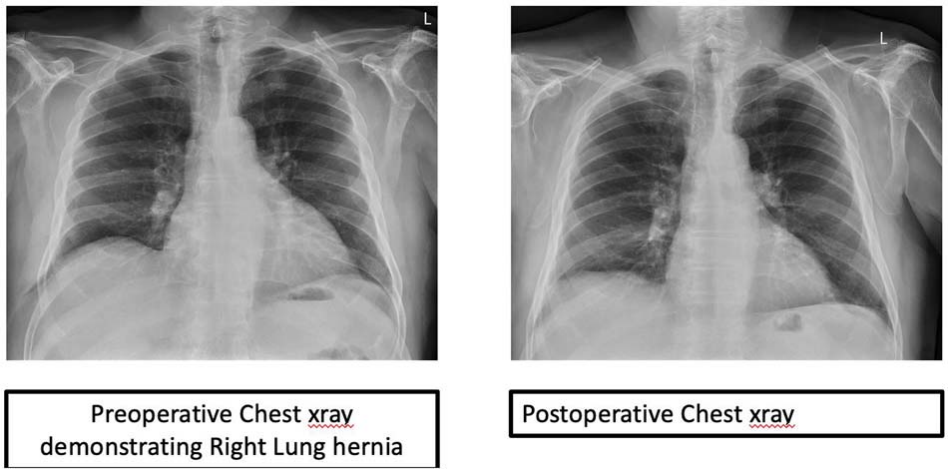
Preoperative and postoperative chest X-rays.

The patient had an uneventful recovery and remains asymptomatic with no recurrence after 6 months (Fig. 3).

## Discussion

Herniation of the pulmonary parenchyma is usually eventuated by trauma or surgery. Spontaneous herniation is rare. Since it was first described by Dr Roland in 1499 [8], very limited numbers have been reported.

The most widely used classification of pulmonary hernia is the Morell Lavellee Classification [9]. This classification differentiates pulmonary hernias based on location and etiology [8].

The biggest risk factors for developing a pulmonary hernia include end-stage COPD or morbid obesity. This appears to be related to the elevated intrathoracic pressure noted in these populations. Conditions or factors that cause poor healing such as diabetes, malnutrition, steroids are also noted as risk factors for developing a pulmonary hernia [10].

Based on the clinical presentation and history of our case, we have classified it as an acquired spontaneous pulmonary hernia according to the Morell Lavellee Classification [2, 5]. Our case presentation is concordant with the classical history noted in the literature with spontaneous herniation of the lung attributed to an increase in intrathoracic pressure. Ongoing swelling and pain necessitated operative repair in our patient. Repair can be by primary closure or through the placement of a prosthetic patch, muscle flap, or fascia lata used to close the defect, mainly when dealing with larger defects which have been described in literature [11, 12].

We opted for primary repair using strong non-absorbable sutures because the defect was easily repaired when the eighth and ninth ribs were brought close by traditional intercostal stitches. We did not feel the need for additional mesh repair.

In conclusion, lung herniation after severe coughing is a rare entity but can become symptomatic and may need surgery. Prompt surgical repair is safe and avoids possibility of subsequent sequelae.
